# Intestinal Stem Cell Niche: The Extracellular Matrix and Cellular Components

**DOI:** 10.1155/2017/7970385

**Published:** 2017-08-01

**Authors:** Laween Meran, Anna Baulies, Vivian S. W. Li

**Affiliations:** The Francis Crick Institute, 1 Midland Road, London NW1 1AT, UK

## Abstract

The intestinal epithelium comprises a monolayer of polarised columnar cells organised along the crypt-villus axis. Intestinal stem cells reside at the base of crypts and are constantly nourished by their surrounding niche for maintenance, self-renewal, and differentiation. The cellular microenvironment including the adjacent Paneth cells, stromal cells, smooth muscle cells, and neural cells as well as the extracellular matrix together constitute the intestinal stem cell niche. A dynamic regulatory network exists among the epithelium, stromal cells, and the matrix via complex signal transduction to maintain tissue homeostasis. Dysregulation of these biological or mechanical signals could potentially lead to intestinal injury and disease. In this review, we discuss the role of different intestinal stem cell niche components and dissect the interaction between dynamic matrix factors and regulatory signalling during intestinal stem cell homeostasis.

## 1. Introduction

The intestinal epithelium is a monolayer of cells covering the entire lumen of the gut that constitutes an important barrier against the external environment. Both small and large intestine share similar glandular crypt structure where intestinal stem cells (ISCs) reside. Crypts are formed by epithelial invaginations into the extracellular matrix (ECM), cushioned by supportive stromal cells. The ISCs, marked by the leucine-rich repeat-containing G protein-coupled receptor 5 (Lgr5), reside at the crypt base alongside their neighbouring Paneth cells [1]. The ISCs divide and give rise to daughter cells entering the transit-amplifying (TA) zone. The TA cells will then proliferate and migrate upwards towards the crypt-villus junction, where they terminally differentiate into all different cell types, including enterocytes, goblet cells, enteroendocrine cells, and tuft cells, before reaching the villus tip and being exfoliated into the lumen, with the exception of Paneth cells that will migrate downwards back to the stem cell zone. The whole ISC proliferation-differentiation journey from the base of the crypt to the villus tip lasts approximately 3–5 days [1–3].

ISCs in the crypt base are maintained by their surrounding niche for precise regulation of self-renewal and differentiation under homeostasis. The ISC niche can be categorised fundamentally into two major components: the “physical” niche and the “cellular” niche. The physical niche refers to the ECM, which comprises an intricate network of fibrous structural proteins (proteoglycans and glycoproteins) that act as scaffolding to maintain the three-dimensional architecture of the intestine. Examples of ECM components surrounding the intestinal crypts include fibronectins, laminin isoforms, collagens, and glycosaminoglycans (GAGs) [4–11]. The cellular niche refers to the stromal microenvironment that comprises all the resident cells embedded within the ECM. These include pericryptal myofibroblasts, fibroblasts, endothelial cells, pericytes, immune cells, neural cells, and smooth muscle cells, which secrete a wide range of matrix components and growth factors for the control of ISC proliferation and differentiation [12, 13]. In addition, Paneth cells constitute another important cellular niche intrinsically within the intestinal crypt. Paneth cells are direct neighbours of LGR5+ stem cells that provide crucial niche factors and signals to support ISC homeostasis [3].

Communication between the ISCs and their niche is regulated by multiple signalling pathways such as the Wnt/*β*-catenin cascade, Notch signalling, Transforming growth factor (TGF-*β*)/bone morphogenic protein (BMP) pathways, and Hedgehog pathways. Perturbations of these pathways or ECM homeostasis due to inflammation, toxins, chemotherapy, and nutritional deprivation can substantially affect the ISC niche, leading to increased susceptibility to intestinal diseases. The ECM is also suggested to act as a reservoir for growth factors via heparin sulfate proteoglycan binding, which may assist in establishing morphogen gradients [14]. These growth factors may also be released upon ECM degradation. In this review, we discuss the contributions of the ECM and cellular microenvironment to the ISC niche and highlight the signalling pathways involved in ISC regulation.

## 2. The Cellular Niche

**Figure 1:**
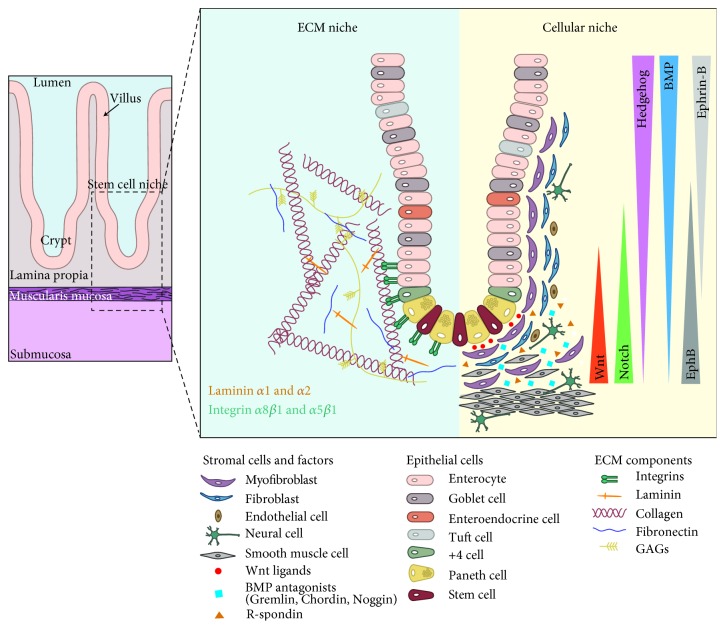
The intestinal stem cell niche. The intestinal epithelium comprises a monolayer of polarised columnar cells organised along the crypt-villus axis. Intestinal stem cells reside at the base of the crypts and continuously generate transit-amplifying (TA) daughter cells that differentiate into various mature cells in the villi (enterocytes, goblet cells, enteroendocrine cells, tuft cells, or Paneth cells). The crypt surrounding microenvironment is made up of both physical/structural and cellular niche to regulate ISC homeostasis. The physical niche includes collagen fibres, integrins, fibronectin filaments, laminins, and glycosaminoglycan, which form a highly structured network named as the extracellular matrix (ECM). The cellular niche includes pericryptal myofibroblasts, fibroblasts, endothelial cells, neural cells, and smooth muscle cells. The ECM and cellular niche interact and communicate with each other via different signalling pathways such as the Wnt, Notch, TGF-*β*/BMP, Eph/ephrin, and Hedgehog pathways for stem cell maintenance.

The mesenchymal compartment of the intestinal lamina propria contains multiple stromal cell populations with distinct phenotypes and function. These include fibroblasts, myofibroblasts, endothelial cells, pericytes, neural cells, smooth muscle cells, and immune cells (Figure 1). The role of intestinal stromal cells in mucosal immunity and homeostasis has been extensively summarised and discussed in several comprehensive reviews, therefore will not be addressed in this review [13, 15, 16]. We will focus on the role of other mesenchymal cells as well as the Paneth cells in ISC homeostasis.

### 2.1. Mesenchymal Cells

*Fibroblasts and myofibroblasts* constitute the major cell components in the lamina propria. Fibroblasts drive wound healing but also have pathological implications in a range of diseases, including carcinogenesis, in various organs. Intestinal subepithelial myofibroblasts (ISEMFs), a member of the fibroblast family, are located in pericryptal regions throughout the lamina propria [17]. TGF-*β* is thought to be a key factor inducing myofibroblast differentiation [18]. ISEMFs play a pivotal role in the ISC niche by secreting crucial factors such as Wnt ligands and BMP antagonists for stem cell maintenance [13, 19, 20]. ISEMFs exhibit characteristics of both fibroblasts and smooth muscle cells with contractile ability. Upon wound healing, an inflammatory response triggers ISEMFs to secrete the matrix metalloproteinases (MMPs) for matrix remodelling [21]. Once the healing process resolves, ISEMFs will undergo apoptosis mediated in part by IL-1*β* [18, 22]. Excessive ECM deposition, on the other hand, is associated with a pathological persistence of activated ISEMFs such as in inflammatory bowel disease [22, 23].

*Smooth muscle cells*, present in close association with ISEMFs, form a thin layer of muscle (muscularis mucosa) to separate the lamina propria from the submucosa. The smooth muscle cells contract and relax to keep the muscularis mucosal layer under constant agitation [13]. This function serves to expel potentially toxic luminal contents out of the crypts and away from the ISC niche. Similar to ISEMFs, smooth muscle cells also express BMP antagonists to repress the differentiative BMP signal and maintain the Wnt activity in the crypt base [20].

*Endothelial cells* present in the lamina propria appear to be important in maintaining epithelial homeostasis. Previous data showed that radiation-induced injury triggered rapid endothelial apoptosis prior to epithelial death *in vivo* [24]. Importantly, loss of epithelial stem cells did not occur when endothelial apoptosis was blocked by basic fibroblast growth factor (bFGF) treatment or by genetic deletion of the acid sphingomyelinase (*ASMase*)—a gene that is required for radiation-induced endothelial apoptosis. Endothelial cells are also implicated in the induction of intramucosal immune responses [16, 25]. Further investigation is required to fully understand their niche role in ISC homeostasis.

*Pericytes* are periendothelial myofibroblast-like contractile cells wrapping around the capillaries, which regulate angiogenesis and capillary wall permeability via paracrine signalling [26]. However, the identity of the pericytes remains controversial regarding their ontogeny and progeny. Distinction between populations of pericytes and myofibroblasts is challenging since they express similar molecular markers [27]. Subsets of pericytes have been reported to be multipotent progenitors that may participate in tissue regeneration [28]. The specific role of pericytes in the ISC niche remains unclear. It is believed that pericytes may function similarly as ISEMFs based on their close developmental origin and identity [26, 27].

*Neural cells* are important for the intestinal epithelial growth. Bjerknes and Cheng showed that enteric neurons participate in the feedback loop that regulates epithelial growth and repair by expressing the glucagon-like peptide 2 (GLP-2) receptor [29]. The enteric nervous system consists of a large number of neurons and enteric glia cells (EGCs) that are interconnected to form the two ganglionated plexuses—the myenteric and the submucosal plexuses. EGCs are located both within the ganglia and in the extraganglionic regions, such as the lamina propria with close proximity to the intestinal crypts [30, 31]. In addition to their neuroprotective function, these mucosal EGCs are thought to play crucial roles in maintaining the intestinal epithelial barrier. Recent data show that EGC homeostasis postnatally is dependent on functional host-microbe interactions, indicating their role in regulating immune responses in the gut [32]. The EGCs also exert protective functions on the intestine by secreting factors such as epidermal growth factor (EGF) and TGF-*β* isoforms following inflammation or injury [33, 34].

### 2.2. Paneth Cells as ISC Niche

The sole importance of the stromal microenvironment as the ISC niche was challenged when ISC-derived epithelial culture was first established in 2009 in the absence of the mesenchymal niche [35]. The study showed that a single Lgr5-expressing ISC was able to grow three dimensionally into crypt-villus budding organoids with full proliferation and differentiation potential in a Matrigel-based culture. The specialised cells intermingled with ISCs at the crypt base—the Paneth cells, are later revealed to provide essential niche signals to their neighbouring stem cells [3]. Paneth cells are regarded as multifunctional guardians of the stem cell niche. They secrete antibacterial peptides such as lysozyme and defensins to sterilise the niche and are crucial for the mucosal defence mechanisms [36, 37]. In addition, they express signalling factors such as EGF, TGF-*α*, Wnt3, and the Notch ligand Dll4, which provide essential trophic support to ISCs [3]. Paneth cell depletion *in vivo* resulted in simultaneous loss of Lgr5+ stem cells, indicating its essential niche role in the gut.

## 3. The Physical Niche: Extracellular Matrix

Separating the mesenchymal compartment from the epithelial compartment is the basement membrane, which consists of two layers: the basal lamina positioned directly beneath epithelial cells and the underlying reticular sheet of matrix that anchors the epithelium to the lamina propria [38]. The basement membrane is a specialised ECM that is jointly produced by both epithelial and stromal cells and is mainly composed of laminins, collagen IV, and fibronectin. The presence of the basement membrane at the epithelial-mesenchymal interface is believed to play a crucial role in regulating epithelial cell homeostasis (comprehensively discussed in previous reviews [17, 39]). In the underlying connective tissue (lamina propria), several specific isoforms of the ECM components such as fibronectins, laminins, collagens, GAGs (e.g., heparan sulfate proteoglycans—also known as perlecan), and integrins are reported to be enriched at the intestinal crypt base, suggesting their potential role in ISC regulation [4–11, 38, 40–42]. A very recent study on matrix reconstitution of the matrix for intestinal organoid culture using minimal essential components provides direct and significant insight into the biochemical and biophysical roles of the ECM in ISC homeostasis [43]. Here, we discuss the role of ECM in the ISC niche through various biological and mechanical parameters (Figure 1()).

### 3.1. Biochemical ECM Roles in the ISC Niche

*Collagen* is the main structural protein in the ECM and is the most abundant protein in our body. The collagen superfamily contains at least 19 different subtypes, with types I, III, IV, and VI uniformly distributed in the healthy intestinal ECM [11, 44, 45]. However, increasing evidence suggests that type VI collagen (which interacts closely with type IV collagen of the basement membrane) is the key regulator for the mechanical microenvironment of the intestinal crypt cells via fibronectin and RGD (Arg-Gly-Asp)-dependent crypt cell interactions [4, 7]. Indeed, intestinal epithelial crypt cells have been demonstrated to secrete type VI collagen into the basal lamina of the intestinal basement membrane [7]. Increases in ECM collagen deposition augment tissue stiffness which alters integrin focal adhesions, growth factor receptor signalling, and acto-myosin and cytoskeletal-dependent cell contractility [46].

*Laminin* is one of the major glycoprotein constituents of the intestinal crypt basement membrane and is recognised to be particularly important in the establishment of epithelial cell polarity [10, 47]. Laminin subtypes are key components of small intestine and colon basement membranes. Laminin *α*1 and laminin *α*2 were shown to be enriched at the crypt regions, while laminin *α*5 was expressed strongly at the villus basement membrane [39, 47, 48]. Laminin *α*5 is believed to play a crucial role in establishing the mucosal pattern of the small intestine by maintaining the villus architecture [48, 49]. The recent study on the designer matrices for intestinal organoid culture has further demonstrated that laminin-111 (*α*_1_*β*_1_*γ*_1_) is important to enhance ISC survival and proliferation [43].

*Fibronectin* is a high molecular weight adhesive glycoprotein found in a wide range of tissues and plays important roles in cell adhesion, migration, growth, and differentiation. Fibronectin contains binding sites for many ECM proteins such as collagens, GAGs, and RGD peptides for cell surface receptors of the integrin superfamily, suggesting its multifunctional role in the ECM [5]. Intestinal fibronectin is secreted by fibroblasts as well as being expressed by epithelial cells and is located throughout the lamina propria [9, 40, 50]. Altered fibronectin deposition patterns are correlated with several intestinal disease states. For instance, upregulation of FN throughout epithelial cells is associated with intestinal fibrosis such as inflammatory bowel disease [5]. Strain forces exerted in the ECM *in vitro* have been shown to induce fibronectin-mediated epithelial cell migration by activating the extracellular signal-regulated kinase (ERK) and myosin light chain (MLC) signalling pathways, indicating the importance of fibronectin in wound closure and epithelial migration [51]. Fibronectin is also postulated to be an activator of the nuclear factor-*κ*B (NF-*κ*B) signalling pathway in the context of intestinal inflammation [5].

*Integrins* are heterodimeric receptors, consisting of *α* and *β* subunits that link the ECM with the intracellular cytoskeleton as part of the RGD-adhesion system, mediating cell anchorage, intracellular signalling, and mechanotransduction [4, 52]. Several integrin subunits and signalling components were previously shown to be expressed at high levels in the ISCs of the *Drosophila* midgut [53]. The study further demonstrated that integrin signalling is required for the maintenance and proliferation of intestinal stem cells but dispensable for multiple lineage differentiation. *β*1 integrins have also been identified as key regulators for ISC proliferation and homeostasis by mediating Hedgehog signalling in a mouse genetic study [54]. The transmembrane *α*5*β*1 integrin receptor has been shown to regulate many fibronectin-dependent biological effects in human tissues [55]. Integrin *α*8*β*1 is another crucial mediator of intestinal crypt cell-matrix interaction via the focal adhesion kinase (FAK) signalling pathway [56–58]. Intestinal epithelial cells have also been shown to be regulated by integrin-linked kinase (ILK) through a fibronectin-dependent mechanism [59]. Overall, these studies suggest an essential role for integrins, in particular *β*1 integrins in promoting ISC homeostasis.

*Glycosaminoglycan* molecules are thought to provide lubrication and structural integrity to cells in the intestinal ECM owing to their high viscosity and low compressibility, thereby providing a passageway between cells to facilitate cell migration [60, 61]. GAGs can function to organise collagen fibre deposition, stimulate angiogenesis, and inhibit coagulation [62]. The specific GAGs of physiological interest in the intestine are heparan sulfate, hyaluronic acid, heparin, and chondroitin sulfate [63]. Heparan sulfate proteoglycan (HSPG) is one of the most well-studied GAGs in the intestine. HSPGs are present in the ECM as linear polysaccharides, which are able to bind Wnt, Hedgehog, TGF-*β*, and FGF proteins in *Drosophila* and *Xenopus* studies [8, 64–66]. Intestine-specific HSPGs are found on the basolateral surface of intestinal epithelial cells and have been shown to promote intestinal regeneration by modulating Wnt/*β*-catenin signalling pathway, suggesting their role in ISC homeostasis [8, 67]. Hyaluronic acid is another chemically simple, high molecular weight, and nonbranching polymer of N-acetyl-glucosamine repeats that exists abundantly throughout the matrix. During disease processes such as in excessive inflammation, these polymers are cleaved to fragments of lower molecular weight that take on signalling roles [68, 69]. Hyaluronic acid binds to CD44, which is expressed on the plasma membrane of many cell types including ISCs [69]. It also binds to the Toll-like receptors TLR2 and TLR4, which are widely distributed in the gastrointestinal tract to mediate the host response to both commensal and pathogenic bacteria [70]. It has been shown that hyaluronic acid administration enhanced intestinal crypt survival of radiation-induced enteritis mediated through TLR4 and cyclooxygenase-2 (COX-2) [70, 71]. Together, the data suggest that GAGs constitute an important niche for ISC homeostasis.

### 3.2. Biomechanical ECM Roles in the ISC Niche

The biomechanical influence of the microenvironment is believed to play important roles in developmental processes, stem cell fate, and lineage determination [72]. Biophysical factors such as cell shape, ECM stiffness, and topography can all contribute to stem cell regulation. Cells perceive physical stimuli via direct contact to the cell adhesion molecules, which allow the cytoskeleton to communicate with the adjacent ECM structures. This enables microenvironmental forces to be sensed and translated into intracellular messages, in a process termed mechanotransduction, to regulate a wide array of physiological processes [73]. The development of *in vitro* technology for the study of the matrix in the past decade has significantly advanced our understanding of the mechanical regulation of stem cell homeostasis. For example, a recent study using intestinal organoid cultures in customized matrices demonstrated that high matrix stiffness enhanced ISC expansion through yes-associated protein 1 (YAP)/Hippo pathway-dependent mechanism, whereas soft matrices promoted differentiation [43]. The Hippo signalling pathway is a key player of the ECM mechanotransduction that controls organ size by sensing the external mechanical forces (discussed in detail in the next section). The downstream key regulator YAP displays nuclear translocation and activation in response to mechanical tension, indicating its importance in cellular mechanosensing and mechanotransduction [74, 75].

In many organs, ECM topography undergoes constant dynamic remodelling whereby components are deposited, degraded, or modified by cues conveyed to the matrix by the surrounding cells [62]. The process of intestinal ECM remodelling is strongly associated with angiogenesis, cell migration, and differentiation as well as tumourigenesis, while ECM deposition and destruction occur via matrix metalloproteinases (MMPs) [76]. MMPs comprise a large family of at least 25 zinc-dependent endopeptidases capable of degrading all components of the ECM. They are classified according to substrate specificity and are associated with human diseases such as rheumatoid arthritis and cancer [77]. Intestinal organoids cultured in RGD-based hydrogels that were susceptible to MMP-mediated degradation demonstrated a proinflammatory phenotype with reduced stem cell maintenance [43]. The findings provide direct evidence that the ECM comprises an essential niche role for the regulation of ISCs.

## 4. Signalling Pathway Regulation in the ISC Niche

The cellular and mechanical niche components in the intestinal crypt communicate with each other via different signalling regulatory pathways to maintain the optimal microenvironment for ISC homeostasis. Here, we discuss the major signalling pathways that are essential for stem cell maintenance and repair (Figure 1()).

### 4.1. Wnt

Wnt signalling is an evolutionary conserved pathway that plays a crucial role for the maintenance and proliferation of intestinal stem cells [78, 79]. Wnt ligands are secreted by various ISC niche cells, including the Paneth cells and the stromal cells surrounding the crypt [3, 80, 81]. Expression analysis in the intestine showed that Wnts 3, 6, and 9b are secreted predominantly by epithelial cells, whereas Wnts 2b, 4, 5a, and 5b are secreted by the mesenchyme [82]. Paneth cell-secreting Wnt3 constitutes the essential ISC niche factor for the stromal-free intestinal organoid culture *in vitro* [3, 83]. Interestingly, Wnt3 deletion or Paneth cell depletion *in vivo* in the gut did not affect intestinal homeostasis, suggesting a redundant role of Wnt ligands from the stromal microenvironment [80, 83].

R-spondin is a potent Wnt agonist that potentiates Wnt signalling in the presence of Wnt ligands via LGR-dependent mechanism [84]. A more recent study further demonstrates distinct, nonequivalent roles of Wnt and R-spondin ligands in ISC homeostasis using lineage tracing mouse models. While Wnt proteins confer a basal competency by maintaining R-spondin receptor expression (LGR4-6, RNF43, and ZNRF3 receptors), they are unable to induce ISC self-renewal and expansion alone *in vivo* without the presence of R-spondin ligands. The data suggest that R-spondin, rather than Wnt, plays the dominant role in controlling the size of the Lgr5+ ISC pool [85]. R-spondin proteins are secreted by the intestinal stromal niche to promote crypt proliferation and ISC maintenance [81, 84, 86]. Indeed, ex vivo stromal cell-free intestinal organoid culture is also dependent on the presence of R-spondin [35]. Depletion of Foxl1-expressing pericryptal mesenchymal cells *in vivo* led to suppression of Wnt activity and ISC proliferation due to the loss of Wnt ligands and R-spondin [87], supporting the important roles of Wnt and R-spondin in ISC maintenance. Similarly, another recent study shows that the CD34+ gp38+ pericryptal mesenchymal cells (also express Foxl1) are the major intestinal source for the ISC niche factors such as Wnt2b, R-spondin, and Gremlin1 [88]. These cells are in close proximity with Lgr5+ ISCs that constitute the key ISC microenvironment by promoting Wnt signalling and antagonising the BMP signalling (see below). On the other hand, several secretory Wnt antagonists such as SFRP-1 and Dkk-3 are also expressed in the stromal cells [82], suggesting the crucial role of ISC stromal niche in controlling the Wnt activity at the “just-right” level for stem cell homeostasis.

### 4.2. BMP

Mesenchymal-derived BMPs belong to the TGF-*β* family. TGF-*β*/BMP signalling inhibits intestinal epithelial stem cell expansion and promotes epithelial differentiation in the crypt [89, 90]. In contrast to Wnt signalling, BMP signals are activated in the villus and are suppressed toward the base of the crypt [89, 91]. Bmp4 is expressed throughout the lamina propria, while the BMP receptor (Bmpr1a) is expressed in the epithelial cells towards the villus [89]. BMP antagonists such as Gremlin1, Gremlin2, and Chordin are secreted by the ISEMFs and smooth muscle cells at the human colonic crypt bottom to repress BMP signalling, while BMP ligands are expressed in the upper colonic crypt to drive differentiation [20]. Similar to human colon, the BMP antagonist Noggin is also expressed at the stromal niche surrounding the crypt in the small intestine [89]. Transgenic expression of Noggin in the intestinal epithelia led to de novo crypt formation [92]. Another secreted protein, angiopoietin-like protein 2 (ANGPTL2), is also expressed by ISEMFs to inhibit *Bmp*2 and *Bmp7* expression via integrin *α*5*β*1/NF-*κ*B signalling and maintain ISC homeostasis [93].

Crosstalk between Wnt and BMP signalling is believed to play a key role in ISC homeostasis. Previous studies showed that deletion of Bmpr1a in mouse intestine caused rapid expansion of the stem cell compartment by enhancing Wnt activity [89]. Recent data further demonstrate that epithelial BMP signalling is crucial to restrict ISC expansion by direct Smad4-mediated repression of Wnt/stem cell signature genes [90]. Importantly, the stromal cells surrounding the intestinal crypt base secrete both Wnt (Wnt and R-spondin ligands) and BMP factors (Bmp antagonists such as Gremlin and Noggin) together to drive ISC proliferation [20, 87, 88]. R-spondin and Noggin also constitute the key growth factors for the stromal cell-free intestinal organoid culture, which can be replaced by coculturing with mesenchymal cells [35, 87, 88]. Together, these findings suggest that ISC stromal cells play an indispensable role for ISC homeostasis by modulating both Wnt and BMP signalling pathways.

### 4.3. Notch

Notch signalling is crucial for ISC maintenance and fate decision, where Notch inhibition resulted in reduced stem cell proliferation [94, 95]. The Notch signalling pathway is regulated through the presentation of the membrane-bound Notch ligand to an adjacent cell expressing the Notch receptor, suggesting the importance of close proximity between ISCs and their niche [96]. Notch receptor and ligand transcripts have been detected in both epithelial and mesenchymal cells of the developing and adult rodent intestine [97–99]. Paneth cells express the Notch ligands delta-like 1 and 4 (Dll1 and Dll4) and present these ligands to their adjacent Notch receptor-expressing ISCs for Notch activation [3]. Simultaneous deletion of Dll1 and Dll4 resulted in loss of ISCs and crypt proliferation, suggesting that Notch activation is required for ISC homeostasis [100]. Activation of Notch has also been shown to be crucial during intestinal epithelial regeneration [101].

Notch signalling is also important in lineage specification at the progenitor cells. Notch activation drives absorptive lineage differentiation, while Notch inactivation drives atonal homolog 1- (*Atoh1*-, also known as *Math1*) dependent secretory lineage differentiation [100, 102–108]. Atoh1 depletion in the intestine resulted in expansion of the crypt proliferative zone and promoted enterocyte over secretory cell differentiation [109]. On the other hand, disruption of Notch signalling caused rapid conversion of all proliferative crypt cells into goblet cells [105, 110, 111]. The ISC-specific marker Olfactomedin 4 (Olfm4) was shown to be a direct Notch target in the intestine [94]. Interestingly, murine Olfm4 has been described as a secreted ECM glycoprotein that promotes cell adhesion and binds to cell surface cadherins and lectins, suggesting a potential link between Notch signalling and the ECM niche [112].

### 4.4. Eph/ephrin

Cell positioning along the intestinal crypt-villus axis is controlled by the Eph/ephrin-mediated interaction and repulsion among the epithelial cells and is crucial for ISC homeostasis [113, 114]. Eph tyrosine kinase receptors and their ephrin ligands are expressed in most adult stem cell niches, often in counter gradients to regulate tissue boundary and stem cell proliferation [115]. *In vivo* studies and gene expression profiling experiments have shown that EhpB2 and EphB3 are both Wnt target genes and are expressed in the proliferative cells at the crypt bottom [113]. Deletion of both EphB2 and EphB3 in mouse intestine altered the positioning of proliferative and differentiated cells and caused mislocation of the Paneth cells scattering along the crypt-villus axis. In contrast to the EphB receptors, ephrin-B1 ligand is expressed in the differentiated cells in an opposite gradient [113]. Interaction of the receptor with its ligand prevents proliferating cells from migrating into the differentiated cell territory, thereby promoting compartmentalisation of the epithelial cells along the crypt-villus axis [113, 116]. In addition to the EphB family, multiple EphA receptors and their ligands are also expressed differentially in human colonic crypts. EphA1, EphA4, and EphA7 are expressed at the crypt bottom, while EphA2, EphA5, and the ephrin-A1 ligand are enriched at the upper colonic crypts [20]. The role of EphA-ephrin-A signalling in ISC homeostasis is yet to be determined. Together, Eph-ephrin signalling is believed to maintain ISC homeostasis by restricting the ISCs and Paneth cells at the crypt bottom for exposure to the key stem cell niche factors.

### 4.5. Hippo

Hippo signalling pathway is highly conserved and plays a central role in organ size control, stem cell renewal, and regeneration via extracellular mechanical forces [117]. The transcriptional coactivators YAP and TAZ have been shown to transduce mechanical cues to mediate biological effects in response to ECM elasticity and cell shape. YAP and TAZ are translocated to the nucleus for transcriptional activation in stiff matrix, while the two effector proteins are excluded from the nucleus in soft matrix [118]. Recent studies suggest an important role of Hippo signalling in regulating intestinal homeostasis and regeneration [119–121]. The effector protein YAP is mainly expressed throughout the intestinal crypt and promotes intestinal regeneration [119]. YAP and TAZ have been shown to induce both proliferation of crypt progenitor cells and differentiation of ISCs into goblet cells via TEADs- and Klf4-mediated transcription regulation, respectively [122]. YAP/TAZ-deletion was also found to impair intestinal organoid formation and prevent Apc loss-induced lethality by Wnt-mediated mechanism [123]. On the other hand, an inhibitory role for YAP in intestinal regeneration has been proposed. Overexpression of a constitutively active YAP-S127A mutant in mouse intestine led to the loss of proliferative crypts and Wnt signal suppression, whereas depletion of YAP in the gut caused hyperactive Wnt signalling and expansion of ISCs and niche cells during regeneration [120]. These paradoxical observations could possibly be explained by the complexity of the Hippo pathway such as cell-ECM interaction, nuclear-cytoplasmic shuttling of YAP/TAZ, and its crosstalk with Wnt signalling cascade. Further investigation on the effect of ECM dynamics to ISC maintenance in the context of Hippo signalling regulation will help understand the mechanical-cytoskeletal cues on stem cell homeostasis and regeneration.

### 4.6. Hedgehog

Hedgehog signalling is involved in stem cell maintenance, organogenesis, and tissue repair/regeneration [124]. Paracrine Hedgehog signalling is crucial for intestinal crypt-villus axis formation during development. Expression of the two ligands Sonic Hedgehog (Shh) and Indian Hedgehog (Ihh) is limited to the intervillus pockets of the developing epithelium, while the expression of the receptors patched 1 (Ptch1) and patched 2 (Ptch2) and the effectors Gli1, Gli2, and Gli3 is restricted to the underlying mesenchyme [125]. Ihh is expressed in the differentiated epithelial cells in the villi of the adult small intestine and is crucial for epithelial integrity and wound healing [126]. Blockade of Hedgehog signalling inhibited villi formation and maintained intestinal crypt proliferation by enhancing Wnt/*β*-catenin activity [125]. Deletion of Shh or Ihh showed multiple gastrointestinal defect and reduced smooth muscle cells [127]. Intestinal-specific deletion of Ihh resulted in disruption of mesenchymal architecture and ECM deterioration via the loss of Bmp signalling and increased MMP synthesis [128]. In addition to regulating smooth muscle and myofibroblasts during development, Hedgehog signalling is also required to induce *Bmp4* expression in the stromal niche to regulate enteric neural cell differentiation [129]. Together, the data suggests that paracrine Hedgehog signalling from epithelial to mesenchymal cells promotes stromal niche formation, which in turn affects epithelial proliferation and differentiation. Hedgehog signalling in the gut represents one of the best examples of the close regulation between ISCs and their niche.

## 5. Conclusion and Future Perspectives

The cellular and ECM niches together constitute a dynamic microenvironment that is critical for intestinal tissue homeostasis. In this review, we provide an overview of the biochemical and mechanical cues originating from the matrix, as well as various vital signalling pathways derived from different cellular niche components that are important for the regulation of ISC maintenance and differentiation. Matrix proteins function in the ISC niche to provide the structural scaffold for maintaining the crypt-villus axis formation, transduce intracellular signalling via integrin binding, and act as a reservoir of growth factors that may be released upon proteolysis. Integrin-mediated stem cell anchoring has been recently shown to be crucial for the maintenance of the stem cell compartment in the epidermis, where human epidermal stem cells express high levels of *β*1 integrins [130]. It will be interesting to further explore the role of the integrin-mediated anchoring mechanism in compartmentalisation of the ISCs and Paneth cells in the intestine apart from the Eph/ephrin signalling.

ECM remodelling can influence the accessibility and the biological cues of the ISC niche. Given the growing evidence of the pivotal role of the microenvironment in inflammatory bowel disease and cancer, ECM components may represent appealing therapeutic targets. Recent studies suggest that epigenetic modification such as histone methylation and acetylation may regulate ISC proliferation and differentiation [131]. Further investigation on the potential link between the microenvironment and epigenetic mechanisms may provide an additional level of stem cell regulation. Recent advances on intestinal tissue engineering further highlight the significance between ISCs and their niche (both physical and biological). A greater understanding of the interplay between different cell populations in the ISC niche and their influence on ECM will shed light on both disease management and regenerative medicine.
